# The impact of hBN layers on guided exciton–polariton modes in WS_2_ multilayers

**DOI:** 10.1515/nanoph-2023-0822

**Published:** 2024-01-23

**Authors:** Ho Seung Lee, Junghyun Sung, Dong-Jin Shin, Su-Hyun Gong

**Affiliations:** Department of Physics, Korea University, Seoul, 02841, South Korea

**Keywords:** exciton–polariton, guided exciton–polariton, self-hybridization, hexagonal boron nitride, transition metal dichalcogenide, tungsten disulfide

## Abstract

Guided exciton–polariton modes naturally exist in bare transition metal dichalcogenide (TMDC) layers due to self-hybridization between excitons and photons. However, these guided polariton modes exhibit a limited propagation distance owing to the substantial exciton absorption within the material. Here, we investigated the impact of hexagonal boron nitride (hBN) layers on guided exciton–polariton modes in WS_2_ multilayers. By integrating hBN layers, we demonstrate a notable enhancement in the quality of guided exciton–polariton modes. The hBN layers can reduce substrate surface roughness and provide surface protection for the WS_2_ layer, mitigating inhomogeneous broadening of the exciton resonance. Consequently, we experimentally observed that the propagation distance of polariton modes substantially increased with hBN layers. Additionally, the polariton spectrum broadened due to efficient exciton relaxation to the polariton states at lower energies. Comparison with simulation data emphasizes that the observed improvements are primarily attributed to enhanced exciton quality. The promising outcomes with hBN encapsulation suggest its potential to overcome strong excitonic losses of the guided exciton polariton in implementing nanophotonic devices. Furthermore, this approach provides a new avenue for exploring the novel physics of guided exciton–polariton modes and their potential applications in polariton-based all-optical integrated circuits.

## 1 Introduction

The emergence of polaritons, which are hybrid quasiparticles resulting from the strong coupling of light and matter, has stimulated a new era of exploration in the field of nanophotonics. Depending on the elementary quasi-particle strongly coupled to photons, polaritons take the form of surface plasmon–polaritons, phonon–polaritons, or exciton–polaritons [1], [2], [3], [4]. In the context of exciton–polaritons, achieving vacuum Rabi oscillations between exciton and photon states demands the integration of a semiconductor quantum structure with a robust exciton resonance into an external cavity structure [5]–[9]. For example, to study exciton–polaritons, semiconductor quantum well structures positioned between two distributed Bragg reflectors (DBRs) have been widely investigated.

Transition metal dichalcogenides (TMDCs) have emerged as a promising platform for strong light–matter interactions, owing to their favorable optical characteristics with a large exciton binding energy [10]–[14]. Recent advancements reveal that the formation of exciton–polaritons in TMDC is achievable without the need for external cavities [15]–[19]. Exciton–polariton modes are evident in suspended TMDC layers even at the monolayer thickness level [4], [15], [20], [21]. On the other hand, TMDC layers on a glass substrate, where the refractive index of the surrounding environment is asymmetric, should be thick enough to have these self-hybridized modes [21]. However, it is crucial to note that exciton–polariton modes in thicker TMDC layers encounter substantial excitonic losses. Consequently, exciton–polariton modes with a high exciton fraction exhibit a considerably shortened propagation length, on the order of a few micrometers [16]–[20], [22]. This limitation presents challenges for their effective implementation in practical nanophotonic applications.

Here, we demonstrate the enhancement of exciton–polariton properties, particularly in reducing propagation losses, by integrating hBN layers with a tungsten disulfide (WS_2_) multilayer. We explored the propagation of guided exciton–polariton modes in multilayer (WS_2_) using three distinct sample configurations: a standalone WS_2_ layer, a WS_2_ layer coupled with a thin hBN layer serving as a buffer, and a WS_2_ layer sandwiched between thin layers of hBN. The investigation focused on the photoluminescence spectra scattered at the sample edges to analyze guided exciton–polariton modes. By altering the distance between pumping spot and detection position, we directly examined the behavior of polariton’s propagation distance. The WS_2_ layer encapsulated by hBN, exhibiting the narrowest excitonic linewidth, demonstrated the most extended propagation of exciton–polaritons. Interestingly, upon analyzing the theoretically calculated *E*–*k* dispersion relation for the three samples, we observed that the influence of the refractive index of the thin hBN layers alone could not account for the observed changes in exciton–polariton properties. Instead, our findings suggest that surface protection and the mitigation of substrate roughness significantly impact the propagation losses of polaritons. Thus, hBN-encapsulated platforms are suitable for unfolding potential in polariton-based nanophotonics in 2D semiconductors.

## 2 Results

### 2.1 Experimental design

**Figure 1:**
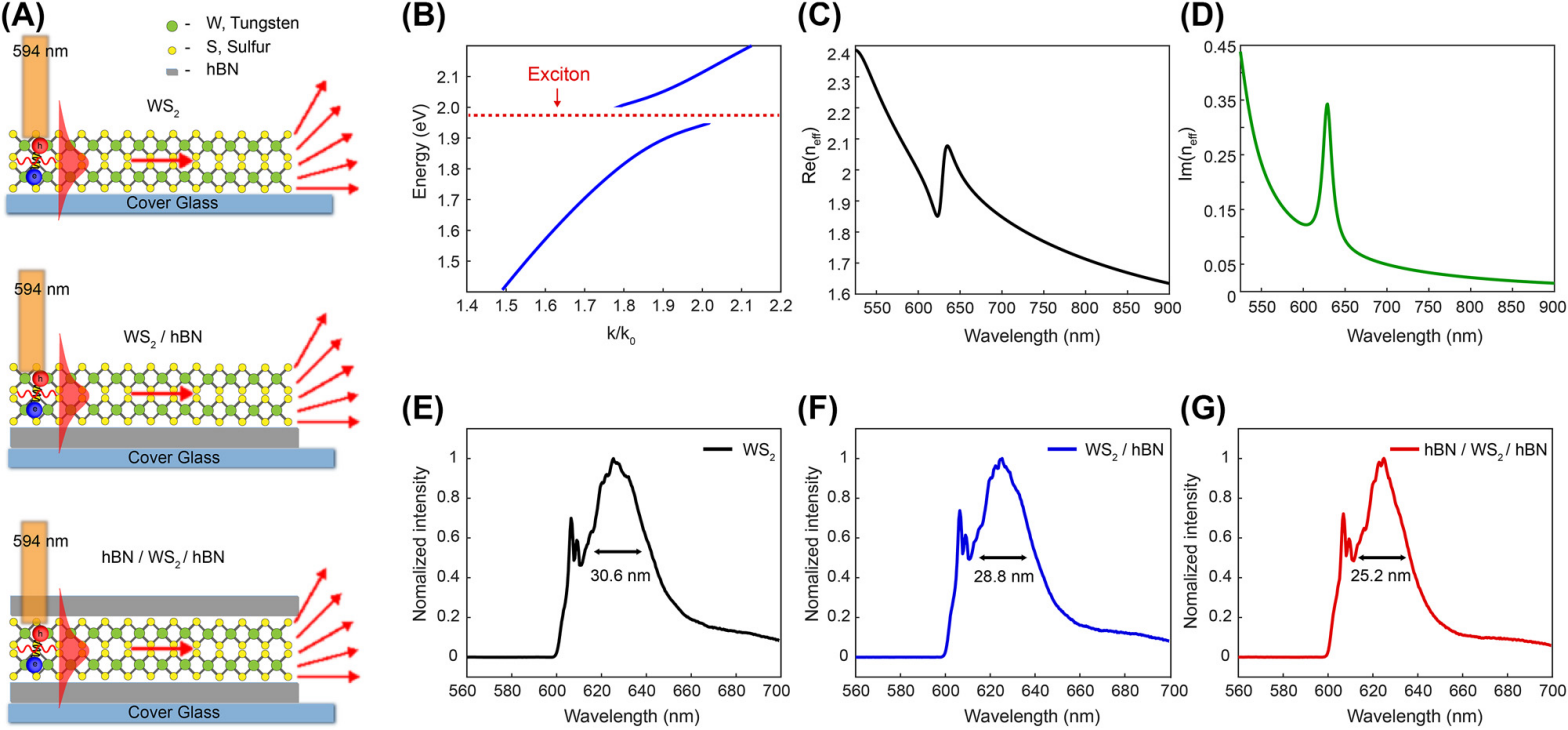
Guided exciton–polariton modes in three different sample configurations: bare WS_2_ layer, WS_2_ layer on top of hBN, and WS_2_ layer sandwiched with hBN. (A) Schematic illustration of guided exciton–polariton modes propagating in the WS_2_ layer without and with the presence of hBN layers. (B) Theoretically calculated optical dispersion relation of the exciton– polaritons. The anticrossing behavior near the exciton resonance (1.98 eV) indicates strong light–matter interaction in WS_2_. (C and D) Real and imaginary effective refractive indices of guided exciton–polariton modes. (E–G) Photoluminescence (PL) spectra of exciton emission from three different samples excited with a 594 nm continuous wave diode laser. The sharp peak around 600 nm corresponds to a laser tail of the excitation laser. The spectra demonstrate the narrowing of the inhomogeneous excitonic linewidth with the presence of an hBN layer.

Figure 1A illustrates the schematic representation of exciton–polaritons modes in WS_2_ multilayers. Among the diverse materials in the TMDC category, we selected WS_2_ due to its remarkable oscillator strength and a high exciton resonance energy of approximately 1.98 eV. Consequently, the WS_2_ layer can sustain robust guided exciton–polariton modes covering a broad spectral range within the visible wavelengths. Notably, for WS_2_ layers positioned on a glass substrate, a thickness above approximately 7 nm is necessary to observe self-hybridized guided exciton–polaritons [16], [20].

Figure 1B displays the calculated exciton–polaritons dispersion curve for a 20 nm-thick WS_2_ layer on a glass substrate. At the energy level of the exciton resonance, anticrossing behavior is observed, indicating a strong light–matter interaction between excitons and photons. Figure 1C and D illustrate the real and imaginary components of the effective refractive index of the exciton–polariton mode in the WS_2_ layer. The real part of the refractive index surpasses that of the glass substrate (*n*–1.45), enabling the confinement and guidance of polariton modes along the WS_2_ layer without direct far-field radiation. On the other hand, the imaginary component of the refractive index exhibits a notably high value near the exciton resonance, attributed to intense exciton absorption. This high imaginary component accounts for the remarkably short propagation distance of the guided exciton–polariton modes.

TMDC layers are notably sensitive to the surface roughness of the substrate. Given the thin nature of TMD layers, the roughness of the substrate induces local potential variations for the excitons, leading to inhomogeneous broadening of excitonic peaks. Therefore, hBN layers, also van der Waals materials, are commonly employed to enhance the optical properties of TMDC layers on substrates. Earlier studies have shown that hBN encapsulation increases the excitonic resonance and diminishes the inhomogeneous excitonic linewidth [22], [23], [24]. The crucial role of the hBN layer lies in mitigating the substrate’s roughness effects and surface protection.

**Figure 2:**
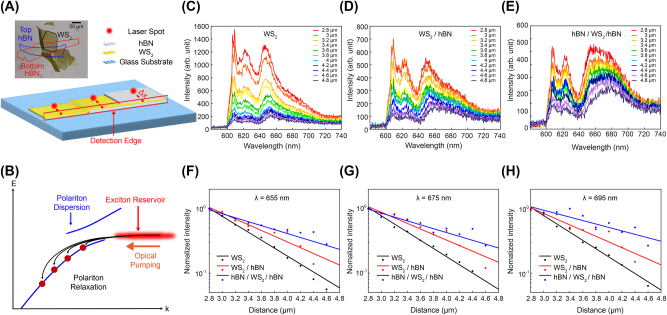
Photoluminescence (PL) measurement of WS_2_ layer with hBN encapsulated structure. (A) Schematic representation of the hBN-encapsulated WS_2_ sample designed for three different measurement positions: bare WS_2_, WS_2_ on top of hBN, and WS_2_ sandwiched with hBN. Inset displays an optical microscope image of the sample with WS_2_ (thickness *d* = 18 nm), bottom hBN (*d* = 15 nm), and top hBN (*d* = 10 nm). Scale bar = 20 µm. (B) Schematic representation of the optically excited exciton in the exciton reservoir relaxing to the polariton states. (C–E) PL spectra of scattered emissions of exciton–polaritons at the edge of the sample with increasing distances between the pumping spot and the edge for three different sample positions. (F–H) The normalized PL intensity as a function of the measured distance at wavelengths of 655 nm, 675 nm, 695 nm, respectively. Scattered plots represent experimental data, and solid lines indicate fitting data for each set of experimental data.

Since the quality of excitons in the TMDC layer directly impacts guided exciton–polaritons, we explored the influence of hBN layers on exciton–polariton modes. We investigated three distinct systems: a WS_2_ layer without hBN, a layer with hBN underneath, and a layer encapsulated by hBN (see Figures 1A and 2A). Before we studied on exciton–polariton modes, we directly investigated the exciton spectrum first, as shown in Figure 1E–G. Each spectrum, whether without hBN or with one- or double-sided hBN layers, exhibited a full width at half maximum (FWHM) of 30.6, 28.8, and 25.2 nm, respectively. These values correspond to linewidth energy of 9.72, 9.21, and 8.11 meV. Notably, the WS_2_ layer encapsulated by hBN showed the most significant reduction in linewidth, by approximately 1.61 meV compared to a bare WS_2_ layer. This confirms that hBN layers effectively mitigate the inhomogeneous broadening of the sample at room temperature.

### 2.2 Photoluminescence measurement of guided exciton–polariton modes

To explore the impact of hBN on guided exciton–polariton modes, we examined the photoluminescence (PL) of these modes under local exciton pumping using a continuous wave (CW) diode laser at a wavelength of 594 nm. The optically pumped excitons in the exciton reservoir can relax to the guided polariton modes through various scattering processes – phonon–, exciton–, or polariton–polariton interactions (see Figure 2B) [25], [26], [27], [28]. However, directly detecting the excited polariton modes using conventional far-field spectroscopy setups is not possible due to the large lateral momentum of these modes (i.e., higher effective refractive index of the polariton modes compared to that of glass). At the sample’s edge, however, where translational symmetry is broken, the polariton modes can undergo scattering to the far-field. By capturing scattered light at the sample’s edge, we investigated the characteristics of polariton emission propagating along the WS_2_ layer.

For this study, we selected a WS_2_ layer with a straight edge and integrated it with hBN layers to observe polariton PL under three different configurations involving hBN layers (refer to Figure 2A). The inset in Figure 2A displays an optical microscope image of the fabricated sample – a WS_2_ multilayer (thickness, *t* = 18 nm) stacked with bottom and top hBN layers (*t* = 15 nm, 10 nm). We aimed to use thin hBN layers in a manner that minimizes the influence of the high refractive index of the hBN layer on the guided polariton modes. The sample was positioned such that the sample edge aligned with the spectrometer entrance orientation. By selectively collecting scattered light at the sample edge using the entrance slit, we ensured a separation between direct exciton emission from the laser pumping spot and polariton emission from the edge. This allowed for distinct observation and analysis of these emissions. Appendix A provides detailed information on the positions of the excitation source and detection edges for each configuration.

Figure 2C–E illustrates the PL spectra of the scattered emission at the sample edge while gradually increasing the distance between a pumping spot and the detection edge. The broader spectrum spanning the spectral range of 640–720 nm represents the polariton spectrum resulting from the relaxation from the exciton state to the polariton states at lower energies (Figure 2B). Notably, the significantly low intensity of the polariton modes at wavelengths shorter than 640 nm is due to substantial propagation losses involving exciton absorption, as estimated from Figure 1D. The observation of the exciton peak at the center wavelength of 620 nm is mainly due to the imperfection of spatial filtering using the spectrometer’s entrance slit, while the peak at the shortest wavelength corresponds to the laser tail.

The primary distinction observed among the three configurations is the spectral bandwidth of the polariton spectrum. The spectrum of polaritons with hBN layers exhibits a broader bandwidth, due to relatively higher intensity in the longer wavelength region. This change is attributed to the polariton relaxation processes enhanced by the presence of the hBN layer. As previously discussed, the integration of hBN results in mitigating surface roughness and provides a surface protection effect, indicating a reduction in nonradiative recombination channels. This reduction leads to a longer exciton lifetime in the exciton reservoir, allowing for sufficient relaxation to the polariton state, even to lower energy levels requiring a longer relaxation time. Therefore, the hBN layer enables the study of polariton emission in lower energy regions.

The second notable difference is observed in the behavior of PL intensity with increasing the distance between the pumping spot and the edge. The intensity exhibits a more gradual decrease at longer wavelengths. Furthermore, at the same wavelength, it diminishes more slowly in the presence of the hBN layer. The emission intensities are plotted against the distance between the pumping spot and the edge at wavelengths of 655, 665, and 675 nm, respectively, in Figure 2F–H. These intensities are normalized concerning the maximum intensity with the shortest measured distance. By fitting a single exponential decay function, the propagation lengths of the polaritons were estimated. At a wavelength of 655 nm, the estimated propagation constants for WS_2_ layer, WS_2_ layer with a bottom hBN, and encapsulated WS_2_ layer are 7.04 μm, 9.87 μm, and 12.9 μm, respectively. Correspondingly, at wavelengths of 675 nm and 695 nm, the estimated propagation constants exhibit values of 7.36 μm, 13.3 μm, 21.6 μm, and 8.74 μm, 11.3 μm, 22.5 μm, respectively. For all measured polariton wavelengths, the presence of the hBN layer facilitates longer polariton propagation. Furthermore, it is noteworthy that hBN encapsulation yields superior results compared to employing just a hBN buffer layer, aligning well with our initial expectations with exciton linewidth results (Figure 1E–G).

### 2.3 The role of hBN layers in guided exciton–polariton modes in WS_2_ layer

As previously mentioned, the high refractive index of the hBN layer can influence the dispersion relation of guided exciton–polariton modes. To discern the role of the hBN layer in these modes, we conducted numerical simulations using the Finite-Difference Time-Domain (FDTD) method. We calculated the *E*–*k* dispersion relation of the polariton mode for three sample configurations: bare WS_2_, WS_2_ with hBN on the bottom, and WS_2_ encapsulated by hBN.

If the excitonic resonance in the WS_2_ layer is modified by the presence of the hBN layer, both the real and imaginary parts of the refractive index of the WS_2_ layer should change according to the Lorentz oscillator model. However, since directly measuring changes in the oscillator strength of the exciton is challenging, we maintained the refractive index of the WS_2_ layer constant. This approach allowed us to distinguish the role of the hBN layer – specifically, the effect of the refractive index of the hBN layer versus the change in the exciton resonance.

**Figure 3:**
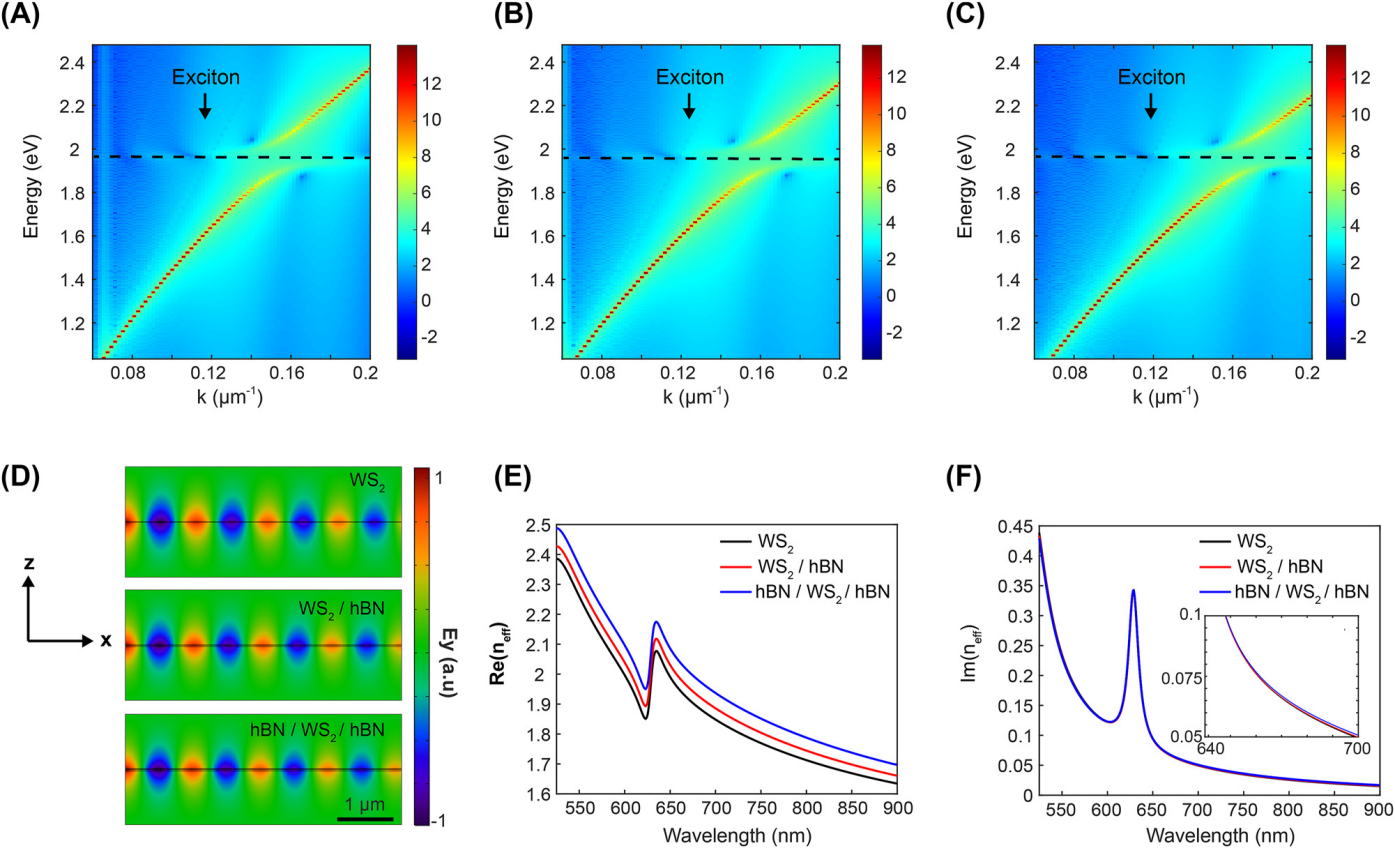
Calculated optical *E*–*k* dispersion relations of guided exciton–polariton modes in WS_2_ layer. (A–C) Simulated results of the dispersion relation for bare WS_2_ layer (A), WS_2_ layer on top of hBN (B), and hBN-encapsulated WS_2_ (C). The energy levels of excitonic resonances are indicated by dotted lines. (D) Calculated electric field profile, *E*_*y*_, of the guided exciton– polariton modes for the three different samples. (E–F) The real (E) and imaginary (F) parts of the effective refractive indices of the exciton–polariton modes as a function of wavelength.

Figure 3A–C illustrate the calculated *E*–*k* dispersion relation of guided polaritons in three samples. Anticrossing behaviors persist at the excitonic resonance energy for all three sample configurations, indicating strong exciton–photon coupling. For a more precise comparison, we plotted the corresponding effective refractive index of the guided polariton modes in Figure 3E and F. Despite using very thin hBN layers with a thickness of 10 and 15 nm, the high refractive index of hBN (∼1.72) results in an increase in the real part of the effective refractive index of the polariton modes (Figure 3E). Meanwhile, the imaginary part of the effective refractive index does not show a significant change because there is negligible optical absorption in the hBN layer (Figure 3F). Nevertheless, the imaginary part also increases very slightly, attributed to the more tightly confined mode distribution in the WS_2_ layer with the hBN layer, leading to higher excitonic absorption in the WS_2_ layer.

**Figure 4:**
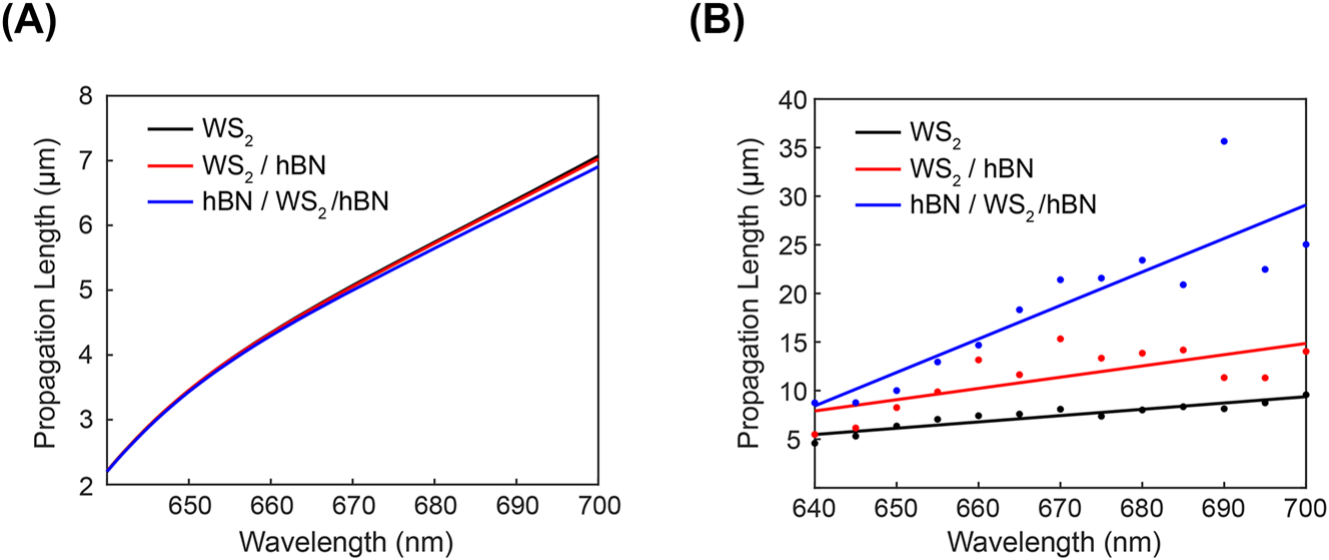
The direct comparison of hBN dependencies on the propagation length of guided exciton–polariton modes. (A) Theoretical calculation of propagation length of exciton–polaritons. When the refractive index of the WS_2_ layer remains constant, minimal differences are observed. (B) Experimental data on the propagation length of exciton–polaritons. Significant distinctions are evident between WS_2_ layer, WS_2_ layer with hBN underneath, and hBN encapsulated WS_2_.

The calculated imaginary refractive index of the polariton mode with and without hBN layers directly relates to the propagation distance constant. Figure 4A shows the calculated propagation distance of the polariton mode in three different sample configurations. Since excitonic losses increase due to the high refractive index of the hBN layer, the theoretically estimated propagation distance becomes slightly shorter with the presence of the hBN layer. However, our experimental results exhibit exactly the opposite behavior.

**Figure 5:**
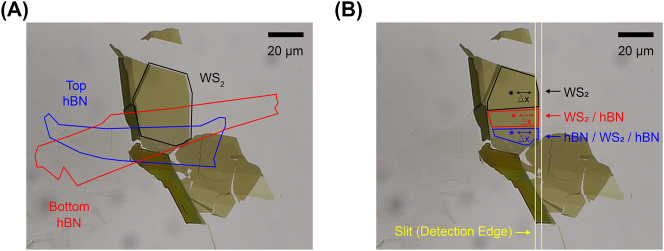
Optical microscope image of the fabricated sample (A) magnified image of the sample with WS_2_, bottom hBN, and top hBN indicated with colored solid lines of black, red, and blue respectively. (B) Optical image of the sample indicating each experimental region of bare WS_2_ layer, WS_2_ layer on top of hBN, and hBN encapsulated WS_2_ layer with colored solid lines of black, red, and blue respectively. The detection edge, where spectrometer slit is placed, is marked with yellow solid lines.

**Figure 6:**
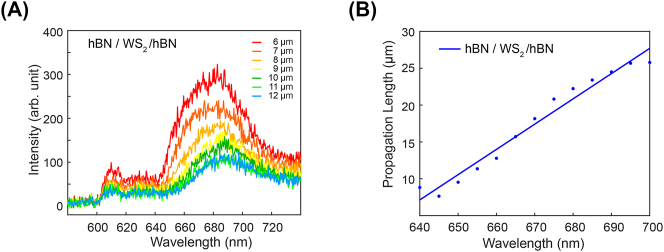
Polariton spectrum with longer travel distance. To further confirm the long propagation length of exciton–polaritons in the hBN-encapsulated WS_2_ layer, we conducted additional measurements with longer propagation distances. (A) PL spectra of scattered emissions of exciton–polaritons at the edge of the hBN-encapsulated WS_2_ with increasing distances between the pumping spot and the edge. (B) Plot of propagation length versus wavelength of exciton–polaritons, which is consistent with the main data in Figure 4.

Figure 4B displays the measured propagation distance as a function of the wavelength. Longer propagation distances were measured with the hBN layer. It is crucial to note that hBN encapsulation increases the propagation distances of guided mode exciton–polaritons by more than twice compared to bare WS_2_ (Figures 5 and 6, Appendix). The long propagation length of the polariton in the hBN-encapsulated sample was additionally validated by measuring position-dependent PL spectra over a long distance, as presented in Appendix B. The contrary trend observed in the experimental results suggests that the primary impact of the hBN layer on the guided polariton mode is the reduction of the inhomogeneous exciton linewidth.

## 3 Conclusions

We have demonstrated a significant enhancement in the quality of guided exciton–polariton modes in WS_2_ layers through integration with hBN layers. The hBN layers play a crucial role in reducing the substrate’s surface roughness and providing surface protection for the WS_2_ layer, thereby mitigating inhomogeneous broadening of the exciton resonance. Consequently, the propagation distance of the polariton modes was greatly increased. Additionally, the polariton spectrum became broader due to the efficient relaxation of excitons to polariton states at lower energies. The hBN encapsulation yielded the best results for the guided polariton modes. Upon comparison with simulation data, we conclude that the experimental results are primarily attributed to the enhanced quality of excitons. We anticipate similar outcomes for guided polaritons in other TMDC layers. We believe that hBN encapsulation resolves the primary obstacle in implementing guided exciton–polariton–based nanophotonic devices. Furthermore, this approach provides a new avenue for exploring the novel physics of guided exciton–polariton modes and their potential applications in polariton-based all-optical integrated circuits.

## 4 Materials and methods

### 4.1 Sample preparation

Multilayer WS_2_ and hBN are mechanically exfoliated from WS_2_ and hBN crystals, which were purchased from the company 2D materials. Encapsulated structures are mechanically stacked with a home-built stamping machine in the sequence of hBN, WS_2_, and hBN layers using a dry-transfer method with a polydimethylsiloxane (PDMS) stamp. The thickness of WS_2_ and hBN are measured with an atomic force microscope (AFM).

### 4.2 Optical spectroscopy setup

Optical measurements were conducted using a home-built microscopy setup with a 100× oil immersion microscope objective lens with NA = 1.45. For photoluminescence measurement, a 594 nm continuous wave laser was used. The spectra scattered at the edge of the sample is measured by placing an entrance slit of a spectrometer (Princeton Instruments, HRS-300, 150 g mm^−1^/800 nm) at the edge of the sample. Precise control over increased distance was achieved using a precision positioning and motion control stage.

### 4.3 FDE and FDTD simulation

Theoretical modeling of effective refractive indices of the polariton modes was done by using a finite-difference eigenmode solver (Lumerical Inc.). The *E*–*k* relations and electric field profile of the polariton modes in WS_2_ layer with and without hBN were simulated by using 3D finite-difference time-domain solver (Lumerical Inc). These simulations were conducted using the refractive index of bulk WS_2_ [30]. The refractive indices of hBN were taken as *n*_*x*_, *n*_*y*_ = 1.84, *n*_*z*_ = 1.72 [31]. For the glass substrate, the refractive index of 1.512 was utilized.
